# Comparison of the Effects of Dry Cupping and Acupressure at Acupuncture Point (BL23) on the Women with Postpartum Low Back Pain (PLBP) Based on Short Form McGill Pain Questionnaires in Iran: A Randomized Controlled Trial 

**Published:** 2017-06

**Authors:** Zahra Yazdanpanahi, Mehrnoush Ghaemmaghami, Marzieh Akbarzadeh, Najaf Zare, Amir Azisi

**Affiliations:** 1 Department of Midwifery, Community Based Psychiatric Care Research Center, Shiraz University of Medical Sciences, Shiraz, Iran; 2 Department of Midwifery, School of Nursing and Midwifery, Shiraz University of Medical Sciences, Shiraz, Iran; 3 Maternal–Fetal Medicine Research Center, Department of Midwifery, School of Nursing and Midwifery, Shiraz University of Medical Sciences, Shiraz, Iran; 4 Department of Biostatistics, Infertility Research Center, School of Medicine, Shiraz University of Medical Sciences, Shiraz, Iran; 5 Department of Traditional Pharmacy, School of Pharmacy, Shiraz University of Medical Sciences, Shiraz, Iran

**Keywords:** Dry Cupping, Acupressure, BL23, Low Back Pain, Postpartum

## Abstract

**Objective:** To evaluate the effects of acupuncture branches on postpartum low back pain severity among the primiparous subjects visiting the selected educational centers affiliated to Shiraz University of Medical Sciences, Shiraz, Iran.

**Materials and methods:** This clinical trial was conducted on 150 (each group of 50 people) cases in 2012. Cupping therapy was done every other day in four 15-20 minute sessions a week. Besides, acupressure was applied according to the circular model for 20 minutes. The patients filled out the short form McGill Pain Questionnaires. Then, the data were analyzed using the SPSS statistical software (v. 16) and repeated measurements and Chi-square tests.

**Results:** In the cupping group, the mean difference of postpartum Low Back Pain intensity reached from 31.8 ± 10.8 before the intervention to 9.0 ± 6.7, 7.5 ± 6.6, and 4.1 ± 3.6 immediately, 24 hours, and 2 weeks after the intervention, respectively and the results of repeated measures ANOVA showed a significant difference between the three follow-up periods (p < 0.05). On the other hand, this measure reached from 31.1 ± 11.0 before the intervention to 22.1 ± 7.3, 16.2±6.0, and 11.7 ± 5.3 immediately, 24 hours, and 2 weeks after the intervention, respectively in the acupressure group.

**Conclusion:** The study results showed that these modalities could sedate the pain. Therefore, they can be applied as efficient treatments for reducing the low back pain.

## Introduction

Low back pain is a very common painful disorder in primary care. In general, 80% of people suffer from this pain at least once in their life and it recurs in 60% of the patients. The symptoms of the disorder are not related to any particular etiological or neurological causes in 85% of the cases and it seems that 23% of the patients suffer from pain for more than 12 weeks and have chronic conditions (1). Low back pain during pregnancy is a highly prevalent syndrome with unknown etiology which is identified by features, such as pain and disability of back and pelvis during pregnancy and the postpartum period. The studies conducted on pregnant women between 1980 and 1990 indicated that more than half of them experienced low back pain at least once during pregnancy and its incidence was reported as 78%. This pain increases as pregnancy progresses and disturbs daily activities, such as carrying things, cleaning the furniture, sitting, and walking, and eventually results in the women's absence from work. Sometimes, it also disrupts sleeping. Yet, its outcomes include disability, increase of dysfunction, and damage to joints and tendons (2). Noren et al. (1997) introduced low back and pelvic pain as a pregnancy-leading disease in Scandinavian countries, which cost over $2.5 billion in 1990 (3-4). General agreement suggests that the climax of the symptoms is within the third trimester between 24 and 36 weeks (5-6). In most cases (93%), it spontaneously disappears after the sixth months of the puerperium period, while chronically continues in other ones (7). Ostgaard (1997) found that the prevalence of back pain would decrease to before pregnancy level up to 2 years after the delivery (18%). Most women consider low back pain as an inevitable part of pregnancy and, consequently, do not seek for treatment. Only 50% go to see a doctor and 70% are recovered. It should be mentioned that most of these women receive more than one type of treatment (8). In addition to conventional medicine, some patients look for an alternative one. For instance, cupping is used by acupuncture specialists and others as a physical treatment (9). Nowadays, cupping is considered as a branch of Complementary and Alternative Medicine (CAM) in treatment of diseases, particularly pain syndromes. Dry cupping simply involves stimulation of the skin by suction and is applied to increase the local circulation of blood and lymph and to relieve painful muscle tension (10). Another branch of acupuncture; i.e., acupressure, is a skill in traditional medicine using fingers to press the key points on the skin surface to stimulate and induce the natural body self-treat abilities (11). In this study, acupoint BL23 (Shenshu) locating symmetrically 1.5 cup lateral to the lower border of the spinous process of the second lumbar vertebra (L2) was selected. According to World Health Organization (WHO) guidelines, most patients have an unpleasant feeling at this point. This point has been used in the treatment of pain syndromes, such as pain and soreness of the lumbar region and knees, icy-cold sensation of the lumbar region and legs, hot and cold sensations of the bones, pain of the genitals, gynecological disorders, such as irregular menstruation and chronic vaginal discharges, and insomnia (12). Studies have indicated CAM to be an effective treatment for reducing back pain during pregnancy. This method is employed by one third of the U.S. population most of whom being reproductive aged women. Thus, it is no surprise to accept it as a favorable treatment for low back pain. In addition, over 90% of prenatal health teams have prescribed some of these non-pharmacological modalities and midwives (93%) had applied them more than doctors (64%) or nurses (57%) (13). considering the large number of the patients in the world and Iran and lack of clinical trials related to cupping in the field of obstetrics and gynecology, it has been attempted to link traditional and routine medicine.

## Materials and methods

**Figure 1:**
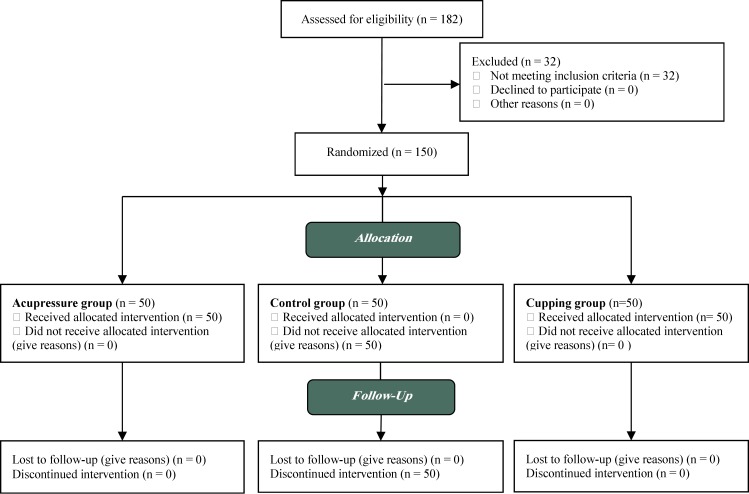
Consort diagram

The present randomized clinical trial was conducted at Hafez Hospital of Shiraz University of Medical Sciences (SUMS), Shiraz, Iran in 2012. In this study, pain diagnosis was based on taking a medical history of the participants. According to the statistical consultation, using the sample size formula (n=(σ12+σ22)(Zα2+Zβ)2D2) the study (N1=N2=N3= 50) (Figure 1) and the results of similar studies and considering the effect size of 2, standard deviation of 1.3, significance level of 0.05, and power of 0.9, a 150-subject sample size was determined for this study. The study samples were selected using simple random sampling. In case the mother's have not met the inclusion criteria of the study, the samples were removed and were replaced by the next one. Then Randomization was performed and were divided into cupping therapy, acupressure, and control groups using stratified block randomization. So, a number was randomly selected from the table of random numbers and the researcher moved toward the right or left column or row and wrote the 5 digit numbers down

Classification was performed as follows: A: cupping group, B: acupressure group and C: control group. Accordingly, ABC: 1, ACB: 2, BAC: 3, BCA: 4, CAB: 5, and CBA: 6. It should be noted that numbers 0, 7, and 9 were ignored. Sampling continues until the number of samples in the same complete. The inclusion criteria of the study were being between 18 and 40 years old, having at least middle school degrees, not having any overt and serious psycho-somatic diseases, such as vertebral fractures, herniated disk, acute inflammation, and deep vein thrombosis, living in Shiraz, being willing to participate in the research project and completing a consent form, and suffering from low back pain merely due to lordosis resulting from pregnancy and childbirth which was intensified by fetus weight and position or labor process in some mothers. Cupping therapy was performed every other day in four 15-20 minute sessions a week. Besides, acupressure was applied according to the circular model for 20 minutes. However, no interventional treatments were performed for the control group. The patients filled out the short form McGill Pain Questionnaires before and immediately, 24 hours, and 2 weeks after the treatments. This instrument is the most reliable measure of pain (particularly chronic and resistant pains) that allows the patients to choose the right words to express their perception of pain (14), but because it will take a long time to complete it by the client to the short form is designed (15). The short form of the questionnaire consists of 15 sensory and emotional items (11 and 4 questions, respectively) and the patients determine the severity of their pain by choosing one of the options of no, mild, moderate, and severe. It is frequently used in Persian Studies (16-17).

Then, the data were analyzed using the SPSS statistical software (v. 16). Repeated measurements test with group and individual adjustment was used in order to compare the changes in mean and standard deviation of pain scores before and after the intervention. Chi-square test was also employed to investigate the demographic characteristics of the three study groups. All the statistical tests were performed considering alpha coefficient of 0.05 and confidence interval of 95%. 

**Table 1:** Demographic Data and Baseline Outcome Values in the cupping, acupressure and control Groups

**Characteristics**	**Intervention groups**		**Control group (n = 50)**
	**Acupressure group (n = 50)**	**Acupressure group (n = 50)**	
Maternal age	25.0, 4.2	25.1, 4.8	27.0, 3.8
Mother's age at marriage	4.2, 22.4	4.8, 23.1	4.4, 22.8
Mother's education	33.33%	32.73%	33.33%
Baseline values			
SMPQ	31.8, 10.8	31.1, 11.0	31.8, 9.8
Sensory SMPQ	22.8, 7.9	22.5, 8.2	23.2, 8.3
SMPQ emotional	9.0, 3.4	8.9, 3.4	9.2, 2.9

* Values are expressed as mean, standard deviation. SMPQ: Short-form McGill Pain Questionnaire

The weak point of this study was that patients were aware of the causes and mechanisms of the interventions and the problem was intensified due to the lack of placebo. However, due to the nature of the procedure, this was out of the researcher's control.

## Results

The study results revealed no significant difference between the three groups regarding the demographic characteristics; i.e., maternal age (p = 0.064), mother's age at marriage (p = 0.210), and mother's education level (p = 0.998) before the intervention. The average pain intensity was 31.8 ± 10.8, 31.1 ± 11.0, and 31.8 ± 9.8 in the cupping, acupressure, and control groups, respectively and the results of ANOVA revealed no significant difference between the intervention groups and the control group (p = 0.1) (Table 1). Immediately after the intervention, the mean and standard deviation of low back pain in cupping, acupressure, and control groups was 9.0 ± 6.7, 22.1 ± 7.3, and 29.2 ± 8.0, respectively and the results of repeated measures ANOVA showed a significant difference between the intervention groups and the control group (p = 0.001). Also, a highly significant difference was observed among the study groups 24 hours and two weeks after the interventions (p < 0.01). Twenty four hours after the intervention, the mean and standard deviation of low back pain in cupping, acupressure, and control groups was 7.5 ± 6.6, 16.2 ± 6.0, and 21.7 ± 6.2, respectively and the results of repeated measures ANOVA revealed a significant difference between the intervention groups and the control group (p = 0.001) (Table 2).

**Table 2:** Means (standard deviations), Mean difference, 95% confidence interval (CI), alpha coefficient of 0.05 and p-value for intervention and control groups

**Measure**	**Phase**	**Intervention groups**		**Control ** **group**	**p-Value**
		**Cupping group**	**Acupressure group**		
SMPQ	baseline	31.8 (10.8)	31.1 (11.0)	31.8 (9.8)	0.1
	Immediately post-intervention	9.0 (6.7)	22.1 (7.3)	29.2 (8.0)	0.001
	24 hrs. post-intervention	7.5 (6.6)	16.2 (6.0)	21.7 (6.2)	0.001
	2 weeks post-intervention	4.1 (3.6)	11.7 (5.3)	14.0 (5.2)	0.001
SMPQ sensory	baseline	22.8 (7.9)	22.5 (8.2)	23.2 (8.3)	0.1
	Immediately post-intervention	7.0 (5.5)	16.4 (5.6)	21.8 (5.9)	0.001
	24 hrs. post-intervention	5.8 (5.4)	12.8 (6.1)	16.4 (3.6)	0.001
	2 weeks post-intervention	3.2 (2.7)	9.2 (4.1)	10.4 (4.0)	0.001
SMPQ emotional	baseline	9.0 (3.4)	8.9 (3.4)	9.2 (2.9)	0.1
	Immediately post-intervention	2.1 (2.1)	5.6 (2.8)	7.5 (2.8)	0.001
	24 hrs. post-intervention	1.7 (1.5)	4.0 (2.4)	5.6 (2.4)	0.001
	2 weeks post-intervention	1.8 (1.2)	2.5 (1.9)	3.6 (1.8)	0.001

Note: SMPQ (Short-form McGill Pain Questionnaire) divided in two parts: sensory and emotional.

## Discussion

The present study was the first clinical trial on the gynecologic diseases in Iran and the world. This study aimed to investigate the therapeutic effects of cupping, suction, and stimulation of different parts of the body by applying pressure on low back pain after delivery. According to the study results and short-form McGill Pain Questionnaire, the intensity of low back pain after delivery decreased from 10.8 ± 31.8 before the intervention to 9.0 ± 6.7, 7.5 ± 6.6, and 4.1 ± 3.6 immediately, 24 hours, and 2 weeks after the intervention, respectively in the cupping therapy group. In addition, the results of repeated measures ANOVA revealed a significant difference among the follow-up stages (p = 0.01). This significant decrease might be due to the physiological effects of cupping therapy which is based on stimulation or relaxation of the body through suction. Up to now, a lot of evidences have supported the use of dry cupping compared to conventional treatment methods for the patients suffering from low back, pelvic, and cancer pains (18).

On the other hand, the intensity of low back pain in the acupressure group reduced from 31.1 ± 11.0 before the intervention to 22.1 ± 7.3, 16.2 ± 6.0, and 11.7 ± 5.3 immediately, 24 hours, and 2 weeks after the intervention, respectively which was significantly different in various follow-up stages. In fact, manipulation and stimulation of small, myelinated peripheral nerve fibers in the muscles using a needle or its substitute; i.e., pressure, send signals from the spinal cord to the midbrain and the pituitary axis eventually leading to release of Encephalin, Dinorphine, and Serotonin into the blood stream as well as the cerebrospinal fluid and reduction of pain (19). This mechanism might have caused the significant reduction in the intensity of low back pain in the acupressure intervention group.

According to short-form McGill Pain Questionnaire, the mean of the sensory dimension of low back pain intensity decreased from 22.8 ± 7.9 before the intervention to 7.0 ± 5.5, 5.8 ± 5.4, and 3.2 ± 2.7 immediately, 24 hours, and 2 weeks after the intervention, respectively in the cupping therapy group and the differences were statistically significant. Based on the cultural effects of cupping therapy which offers a clear explanation about the cause and treatment of the disorder, the disease is hidden in the blood. When blood is accumulated at one point, the disease disappears and the patients feel that the disease is leaving their bodies (20). Of course, the researchers believe that it might have psychological effects, as well.

In the acupressure intervention group, on the other hand, the corresponding values were 22.5 ± 8.2, 16.4 ± 5.6, and 12.8 ± 6.1, respectively which were not statistically significant compared to those of the cupping therapy group. However, a significant difference was observed within the acupressure group 24 hours and 2 weeks after application of pressure compared to before the intervention. This might have resulted from the role of acupressure in releasing lactic acid and carbon monoxide accumulated in the body while muscles contraction which helps the patients’ satisfaction (21). On the other hand, the insignificant difference might be due to the patients’ discomfort by the researcher’s finger pressure on one point which interferes with statement of the intervention’s benefit in reducing the sensory dimension of pain.

In the cupping therapy group, the mean of the emotional dimension of low back pain intensity based on the short-form McGill Pain Questionnaire decreased from 9.0 ± 4.3 before the intervention to 2.1 ± 2.1, 1.7 ± 1.5, and 1.8 ± 1.2 immediately, 24 hours, and 2 weeks after the intervention, respectively and the difference among the follow-up stages was statistically significant. However, this value increased after 2 weeks compared to 24 hours after the intervention which might have resulted from the disruption of the relationship between the mothers and the researcher. In fact, the psychological effects of cupping therapy result from the sequential status of the treatment. Thus, the close relationship between the cupping therapist and the patients plays a critical role in the emotional dimension of pain (22).

In the acupressure intervention group, the corresponding values were 8.9 ± 3.4, 5.6 ± 2.8, and 4.0 ± 2.4, respectively which were significantly different in comparison to those of the cupping therapy group. Nonetheless, a significant difference was observed in the acupressure intervention group 24 hours and 2 weeks after the intervention compared to before the intervention. In addition to the pain reduction mechanism of acupressure and increase of mothers’ tranquility, continuous presence of the researcher by the patients during the intervention as well as providing them with the necessary directions and following them up until two weeks after the delivery have been effective in improving the patients’ emotional status in perceiving the intensity of low back pain. Similar conditions were also observed in the study Kim et al (2011) conducted in order to determine the effect of cupping therapy on reduction of low back pain. That randomized clinical trial was performed on 21 patients in the cupping therapy group and 11 ones in the control group who had been suffering from low back pain for at least three months. The participants were randomly assigned to either the cupping therapy or the control group. The intervention group underwent cupping therapy on BL23, BL24, and BL25 points for 6 times in 2 weeks. On the other hand, the control group received exercise brochures and general recommendations. It should be mentioned that both groups were allowed to use acetaminophen. The study instruments included a 0-100 Numerical Rating Scale (NRS) for pain, McGill Pain Questionnaire for Pain Intensity (PPI), and Oswestry Disability Questionnaire (ODQ). Before the intervention, McGill Questionnaire showed the mean score of pain to be 43.2% in the cupping therapy and 91.1% in the control group and the difference was not statistically significant. After the intervention, however, the mean score of pain was reported as 2.1% and 7.1% in the cupping therapy and the control group, respectively and the difference was statistically significant (p < 0.01). In addition, acetaminophen consumption was significantly lower in the cupping therapy group during 4 weeks (p = 0.09). On the other hand, 6.5 and 8.1 points reduction was observed in the ODQ scores of the cupping therapy and control groups, respectively which was not statistically significant (p = 0.14). Overall, since the reported pain intensity had reduced for 30%, cupping therapy on BL23-25 points can be considered as an effective method in reducing the low back pain but the reason is unknown (23). Similarly, Kim conducted a pilot study in order to investigate the effect of dry cupping therapy and used bladder circuits for placing the cupping. These circuits are related to the waist and application of pressure or cupping on the circuits will be effective in reducing the back pain. In that study also, cupping therapy lasted for 2 weeks but it was performed for 6 times. In contrast to Kim’s study, the present one used the short-form McGill Pain Questionnaire. Moreover, in spite of the similarity of the pain intensity before the intervention, it reached the significance level after the intervention, which is in line with the findings of the current study.

Furthermore, Yen et al (2004) carried out a randomized, controlled clinical trial in an orthopedic specialty hospital in Taiwan. In that study, 146 subjects who suffered from chronic low back pain were randomly divided into an acupressure (n = 69) and a physical therapy group (n = 77). A self-report in the form of short-form McGill questionnaire was obtained from the patients before and after the intervention. The study results revealed no significant difference between the two groups regarding the basic characteristics (p = 0.13) (24). Before the intervention, no significant difference was found between the two groups regarding McGill questionnaire score. Although Yen compared acupressure to physical therapy, the superiority of acupressure can be observed compared to both conventional and new medicine.

In the same line, Yeh et al (2012) investigated the effect of auricular acupressure treatment on chronic low back pain in the U.S. The intensity of pain was measured using the short-form pain questionnaire after 7 days of intervention. The patients in the worst possible pain and moderate pain groups respectively reported 46% and 50% reduction in their pain intensity at the end of the intervention. Overall, the researchers concluded that acupressure could help treat the back pain through the body’s meridians (25).

Considering the fact that cupping therapy has been widely used for treatment of low back and pelvic pain in Korea and the cupping therapist have mostly been female, the survival of cupping therapy may result from the societies’ social, cultural, and economic features.

One other study supporting the basic principles is the randomized, controlled clinical trial performed by Yip et al (2004) in Hong Kong in order to assess the effect of stimulation of the pressure point with electrodes together with acupressure. One week after the treatment, the intervention group demonstrated 39% greater pain reduction on VAS compared to the control group (p = 0.0001) (26).

In Germany, Lauche et al (2011) conducted a pilot study and investigated the effectiveness of dry cupping therapy as an old method for treatment of painful syndromes in the patients suffering from chronic neck pain. In that study, the patients’ sensorimotor threshold, self-evaluating scale of pain outcomes, and life quality were evaluated. The intervention group underwent cupping therapy for 5 times during 2 weeks. Consequently, the intervention group’s neck pain decreased every day and a significant difference was observed between the two groups’ reported pain after the intervention (p = 0.001) (27). Traditional medicine, particularly cupping therapy, originated from the eastern parts of the world; nevertheless, Lauche managed to develop this technique to the European societies.

Overall, the findings of the studies conducted on the issue support the use of traditional medicine in treatment of diseases and up to now, they have been accompanied by no severe complications to affect their efficiency and safety.

***Conclusion:*** The results of the present study showed that although the pain intensity decreased in both study groups, the reduction of pain intensity was significant in the cupping therapy group. Therefore, both cupping therapy and acupressure can be effective in reduction of postpartum low back pain in primiparous women.

Apparently, placebo should be used in order to avoid bias. However, it was not employed in the current study due to the transparent nature of its performance. Thus, further clinical trials using placebos are needed to be conducted in order to determine the accuracy of the obtained results. Moreover, safety is of great importance in order to evaluate the efficiency. In wet cupping therapy, sterilization was not completely observed resulting in the risk of blood borne infectious diseases through contaminated instruments. However, this limitation cannot be attributed to the present study since it utilized dry cupping therapy.
